# Chronic Conditions and School Participation of First-Year University Students—HOUSE ULisbon Study

**DOI:** 10.3390/children9091397

**Published:** 2022-09-15

**Authors:** Ana Cerqueira, Fábio Botelho Guedes, Alexandra Marques-Pinto, Amélia Branco, Cecília Galvão, Joana Sousa, Luis F. Goulao, Maria Rosário Bronze, Wanda Viegas, Tania Gaspar, Emmanuelle Godeau, Margarida Gaspar de Matos

**Affiliations:** 1 Institute of Environmental Health (ISAMB), Faculty of Medicine, University of Lisbon (FMUL), 1649-028 Lisbon, Portugal; 2 Faculty of Human Kinetics, University of Lisbon/FMH-UL, 1499-002 Lisbon, Portugal; 3 Research Center for Psychological Science, Faculty of Psychology of the University of Lisbon, 1649-013 Lisbon, Portugal; 4 GHES Research Center—Office of Economic and Social History, ISEG—Lisbon School of Economics & Management of the University of Lisbon, 1249-078 Lisbon, Portugal; 5 Institute of Education, University of Lisbon (IEUL), 1649-013 Lisbon, Portugal; 6 Nutrition Laboratory, Faculty of Medicine, University of Lisbon, 1649-028 Lisbon, Portugal; 7 Linking Landscape, Environment, Agriculture and Food (LEAF) Research Unit, School of Agriculture, University of Lisbon (ISA/ULisbon), 1349-107 Lisbon, Portugal; 8 Faculty of Pharmacy, University of Lisbon (FFULisbon), 1649-003 Lisbon, Portugal; 9 Institute of Experimental Biology and Technology (iBET), 2780-157 Lisbon, Portugal; 10 Digital Human-Environment Interaction Labs (HEI-LAB), Lusófona University of Humanities and Technologies, 1749-024 Lisbon, Portugal; 11 French School of Public Health, EHESP, 35043 Rennes, France; 12 CERPOP—UMR 1295, Unite Mixte UMR INSERM—Université Toulouse III Paul Sabatier—Team SPHERE, 31000 Toulouse, France; 13 APPSYci, ISPA—University Institute, 1149-041 Lisbon, Portugal

**Keywords:** chronic conditions, university students, time of diagnosis, school participation

## Abstract

Students with chronic conditions (CC) tend to experience several barriers in terms of their school participation and performance. Therefore, the present study aims to explore the factors related to the time of diagnosis of CC (recent/non-recent), the barriers to participation and academic success (health condition, people’s attitude towards CC and school physical environment), the physical and mental health (physical/psychological symptoms and concerns) and school-related variables (relationship with teachers and peers), regarding the school participation of first-year students with CC. This work is part of the HOUSE-Colégio F3 Project, University of Lisbon, which includes 1143 first-year university students from 17 Faculties and Institutes of the University of Lisbon. In this specific study, only the subsample of 207 students with CC was considered, 72.4% of which were female, aged between 18 and 54 years (*M* = 20.00; *SD* = 4.83). The results showed that students with a recent diagnosis of CC and students with school participation affected by the CC were those who presented more negative indicators regarding barriers to school participation, physical and mental health, and school-related variables. A greater impact of CC in terms of school participation was associated with having a recent diagnosis, with people’s attitude towards CC and with the health condition as barriers, with more psychological symptoms and worse relationships with teachers and peers. This is a relevant message for the organization of health services for students with CC at the beginning of their university studies, especially since they are often displaced from home and managing their health conditions alone (in many cases, for the first time).

## 1. Introduction

Chronic diseases or non-communicable diseases are long-term health conditions with a generally slow progression. These conditions are not transmissible from person to person and are characterized by the need for monitoring and management throughout the life cycle [1,2]. Cardiovascular, oncological, respiratory diseases, and diabetes are some of the examples of health conditions that fall within the spectrum of chronic conditions (CC) [3]. 

A diagnosis of CC confronts individuals with a new reality, full of challenges and emotionally stressful situations (e.g., physical symptoms, medical treatments, hospital admissions, possible changes in the family environment), which vary depending on the type of health condition [4,5,6,7]. The need to adapt to a new health condition can lead to situations of stress, anxiety, and depression, among other problems. Thus, CC can constitute a risk factor for the development of mental health problems [8,9,10,11,12,13]. Furthermore, evidence points to the existence of an increased risk in students with CC to experience difficulties in terms of adjustment and psychosocial functioning throughout childhood and adolescence. This scenario also tends to occur in early adulthood [9,14,15,16].

The transition to higher education is itself a phase of several changes and challenges (e.g., academic environment and expectations, interpersonal relationships/social support) that can become more complex when associated with the experience of a CC [11,15,17,18,19,20,21]. Students with CC tend to experience more academic difficulties compared to their peers, which can be reflected in the psychosocial results obtained later in adulthood [22]. The type of chronic health condition that exists influences the level of school participation and academic success. It is crucial to explore the protective and risk factors in these domains [22,23] since the school participation of students with CC can be reflected in their development and psychosocial well-being [23,24,25,26]. 

The students’ school connection and involvement (which includes the relationship with peers, teachers and school community in general) is related to their health, academic, and behavioral results [27,28]. Similarly, negative relationships between teachers and students can be a risk factor for their psychological adjustment. The relationships that are established between young people with CC and their peers have also a decisive weight in their lives and can represent a source of support or of stress. There is evidence that negative peer relationships result in compromises to the students’ psychological well-being. Students with CC may experience more difficulties in social integration with their peer group. At the same time, their ability to establish or maintain these relationships may be compromised, especially in situations of school absenteeism due to their health conditions [28]. 

A more supportive school environment is associated with better results and more positive school experiences for students with CC. Greater support from teachers tends to promote greater school involvement of students with CC [29]. On the other hand, peer support is also important for these students [20]. Thus, the experiences and school participation of students with CC tend to be influenced by the way their peers behave towards them (e.g., students with CC can suffer situations of social isolation and be victims of bullying), by the possible high degree of school absenteeism resulting from the specificities of their health condition and by the lack of adequate support to fulfill their needs [28,30,31,32,33]. 

Young people with CC tend to experience several barriers to their participation and academic success, such as the impact of the disease itself, absenteeism, and the expectations of parents and teachers [22]. It is not only the management of the health condition-related physical aspects that pose barriers to students with CC. They also need to deal with the impact that the CC can have on their psychological, emotional, and social functioning [34,35].

A healthy school environment includes factors such as the physical environment (i.e., at school and in the surrounding space), the psychosocial environment, safety, and school culture. Evidence in the literature points to a relationship between the physical and social school environment and academic performance, attendance, dropout rates, behavior problems, cognitive skills (e.g., concentration and memory), and mood [36,37]. Although the barriers related to the physical environment constitute a risk factor for the school participation of these students, the barriers related to the attitudes and institutional factors are the ones that are most frequently reported in the literature [38]. Thus, the behavior and attitudes toward CC on the part of peers, teaching and non-teaching staff, and parents could explain the results obtained by the students with CC [39,40,41].

All the specificities associated with different health conditions often lead to situations of decreased academic performance, school absenteeism, and feelings of “being different” compared to peers [28,42]. Thus, CC can also have an impact on stigmatization, in the sense that certain characteristics associated with the disease or, in some cases, the lack of visible signs of the disease, may contribute to the stigmas associated with these health conditions. These stigmas cause psychological and social damage [43,44,45]. Considering the weight that a diagnosis of CC has in the students’ lives as well as the associated barriers to their school participation and, consequently, to their psychosocial well-being (that can last through adulthood), this study aims to explore factors related to the time of diagnosis of CC (recent/not recent), barriers to participation and academic success (health condition, people’s attitude towards CC and school physical environment), physical and mental health (physical/psychological symptoms and concerns) and school-related variables (relationship with teachers and peers), with regard to school participation of students with CC. It also intends to analyze the differences between students with a recent/non-recent diagnosis of CC and with/without school participation affected by the CC in the remaining mentioned variables (i.e., barriers, physical and mental health, school and time of diagnosis in the case of the differences related to school participation affected by the CC).

To the best of our knowledge, studies on the school participation (and associated barriers) of first-year university students with CC are scarcer compared to those that focus on the younger population (i.e., children and adolescents). Thus, the present study intends to:

▪ Extend prior research and contribute to increasing the knowledge regarding this specific population (i.e., first-year university students with CC);
▪ Deepen the impact that the time of diagnosis can have on the students’ psychosocial functioning;
▪ Explore more in-depth the type of barriers that students with CC feel in terms of their school participation;
▪ Contribute to health, school, and public policies regarding the importance of adequate health care for university students with CC since first-year students experience a decisive phase in the management of their health condition (they are often displaced from home and manage their health conditions alone, in many cases for the first time) and in the transition from pediatric care to adult care.

## 2. Method

This work is part of the HOUSE-Colégio F3 Project, University of Lisbon, promoted by the Colégio F3, University of Lisbon, which aimed to explore the health and lifestyle profile of 1st year students at the University of Lisbon.

The study was approved by the Ethics Committee of the Academic Medical Center of Lisbon of the Lisbon North Hospital Center, EPE (Ref. 156/20—7 July 2020). All the respondents participated voluntarily. The questions used in this specific study were based on the Health Behavior in School-Aged Children study questionnaire (HBSC) [46,47]. The answers to the questionnaire were obtained online and anonymously, having as an inclusion criterion being a 1st year student at the University of Lisbon. More details on the data collection procedures of the HOUSE-Colégio F3, Ulisbon study can be found in the final report [48].

### 2.1. Participants

The study included 1143 1st year university students from 17 Faculties and Institutes of the University of Lisbon, of which 771 (67.5%) were female, aged between 17 and 65 years (*M* = 19.62; *SD* = 3.96). In this specific study, a subsample of students with CC was considered, which corresponds to 207 participants, 72.4% (*n = 147*) of whom were female and aged between 18 and 54 years (*M* = 20.00; *SD* = 4.83). Of the students with CC, 28.7% (*n* = 50) had a recent diagnosis (in the last two years) and 23.7% (*n* = 46) reported that their school participation was affected by the existent CC. 

### 2.2. Measures and Variables

Considering the objective of the present study, the variables presented in Table 1 were considered. It should be noted that the term school refers to “a place or institution where people receive instruction” [49] (p. 939). Thus, in this study, it is used as a general term that characterizes the participants’ formal education (including university). 

### 2.3. Data Analysis

Data analysis was performed using the SPSS statistical program, version 25 for Windows. Descriptive statistics were performed for all variables. The chi-square test for independent variables was used to analyze the relationship between recent diagnosis and school participation affected by the CC, concerns and barriers to participation and academic success (i.e., school physical environment, people’s attitude towards CC and the health condition itself). The independent samples *t*-test was used to analyze the relationship between recent/non-recent diagnosis and school participation affected by the CC, physical and psychological symptoms, relationship with teachers and peers.

Finally, a linear regression model (adjusted to age and gender) was developed to analyze the predictive value of the different variables under study in the explanation of the school participation affected by the CC. A significance level of *p* < 0.05 was determined.

## 3. Results

The participants are characterized in Table 2 as well as the analysis of their differences regarding the time of diagnosis of CC (i.e., recent/non-recent). According to the results, it was possible to observe statistically significant differences only for three variables under study: health conditions as a barrier to participation and academic success, relationships with peers, and school participation affected by the CC. Students with a recent diagnosis of CC reported their health condition as a barrier to their participation and academic success. In addition, these students also reported a greater impact of the CC in terms of school participation (i.e., attendance, participation, and academic success) and a worse relationship with their peers.

Table 3 presents the bivariate analysis of the differences between students with and without school participation affected by the CC. Statistically significant differences were found for some of the variables under study: recent diagnosis, people’s attitude towards CC, and the health condition itself as barriers to participation and academic success, physical symptoms, and relationship with peers.

The results showed that students with school participation affected by the CC have a recent diagnosis and identify more barriers to their school participation (i.e., people’s attitude towards their health condition and the health condition itself). They are also the ones who have more physical symptoms and a worse relationship with peers. 

The multiple linear regression model presented in Table 4 intends to explore the impact of variables related to the time of diagnosis, barriers to school participation (i.e., health condition, people’s attitude towards CC and school physical environment), physical and mental health (i.e., symptoms and concerns) and school-related variables (i.e., relationship with teachers and peers), with regard to school participation affected by the CC. The model includes the variables used in the bivariate analysis, was adjusted to gender and age, *F* (11,151) = 17,99; *p* ≤ 0.001, and presented a variance value of 55.3%.

According to the model, the school participation affected by CC is positively associated with the recent diagnosis of CC, people’s attitudes toward CC, and health conditions as barriers to school participation. Additionally, school participation affected by the CC has a negative relationship with psychological symptoms and with the relationship with teachers and peers. This means that school participation affected by the CC is associated with more psychological symptoms and worse relationships with teachers and peers.

## 4. Discussion

This study aims to analyze the differences between students with recent/non-recent diagnosis of CC and with/without school participation affected by the CC, regarding variables related to the barriers to school participation (i.e., health condition, people’s attitude towards CC and school physical environment), physical and mental health (i.e., physical/psychological symptoms and concerns), school-related variables (i.e., relationship with teachers and peers) and time of diagnosis (in the case of the differences related to school participation affected by the CC). It also intended to explore factors related to the time of diagnosis of CC, barriers to school participation, physical and mental health, and school-related variables, with regard to the school participation of students with CC.

The results show that students with a recent diagnosis of CC and students whose school participation is affected by the CC are those who present more negative indicators. Thus, students with a recent diagnosis are the ones who report a greater impact of the CC on their school participation (i.e., attendance, participation, and academic success). These are also the students who feel that their health is a barrier to their participation and academic success and who have a worse relationship with their peers.

Being diagnosed with CC is usually an unexpected moment and there are several possible reactions, which can range from worry, fear, despair, anger, and revolt. The way in which the diagnosis is seen influences the acceptance or denial of the situation, tending to also influence the individuals’ functioning and psychosocial outcomes [51]. This is in line with the results obtained in this study. Thus, it is common to have adjustment difficulties in the phases closest to the diagnosis of CC, which can be reflected in emotional or behavioral problems. Difficulties at this stage can also represent an increased risk in terms of mental health [9,10,12]. The importance of an adaptation to the CC that is made over time is highlighted, in order to integrate the new aspects that come from the health condition and that can change the notion of identity, interpersonal relationships, and even future plans [52]. 

Regarding the variables associated with the school participation of students with CC, the results show that a greater impact of CC in terms of school participation is associated with the recent diagnosis, with people’s attitude towards CC and with the health condition as barriers, with more psychological symptoms and worse relationship with teachers and peers. The results obtained in this study are in line with the literature insofar as evidence points to the fact that CC is likely to affect students’ participation [22] which in turn affects their quality of life and psychosocial well-being [23,24,25,26,53].The physical, psychosocial, and organizational dimensions of the different school contexts influence the health and well-being of all its members. The physical and psychosocial characteristics of this environment are factors likely to affect students’ performance and school participation [37]. Like the results obtained in this study, the literature points to the fact that students with CC tend to experience more barriers at a physical, social, and institutional level in the different contexts of their lives (e.g., school) [54].

The literature also suggests that the stigma perceived by individuals with CC is associated with several negative indicators related to interpersonal relationships, quality of life, and psychosocial functioning [43,55,56]. This is in line with the results of this study regarding the barriers to school participation related to people’s attitudes towards CC. Greater functional limitations as well as discrimination felt by others are factors that are reflected in higher levels of perceived stigmatization [43]. 

The decision to share with others (i.e., family, peers, peers) the diagnosis of CC tends to be a difficult decision for young people. This decision can cause doubt, insecurity, and fear regarding the consequences of this sharing and the anticipation of potentially associated stigma. These factors will consequently be reflected in their psychosocial well-being. The main reasons that lead young people to decide not to share their diagnosis with others are fear of rejection and/or fear of being treated with pity as well as the perception of being seen as vulnerable or different [35].

With regard to mental health, the results of a study by Ferro et al. [9] show that CC are associated with the presence of more depressive symptoms over time (i.e., between adolescence and early adulthood). Thus, the results obtained in this study are in line with the literature in the sense that the existence of a CC increases the vulnerability of individuals with regard to mental health problems [8,9,11,13,57],. In the same sense, a study by Herts et al. [11] with first-year university students revealed that students with CC have higher levels of loneliness and isolation and lower levels of quality of life compared to their peers.

Another important aspect regarding the school participation of students with CC concerns the quality of the relationships that are established in this context and that can influence the development of students, insofar as negative experiences are reflected in worse academic and psychosocial results [27]. Evidence points to the relationships between students–teachers and students–students as factors related to psychological adjustment, mental health, and school involvement. Thus, the social relationships experienced by students regarding the school context can be a risk or a protective factor for their psychosocial development, mediated by aspects such as absenteeism, stigmatization, and exclusion related to participation in activities [28]. 

The characteristics of the school context are reflected in the results obtained by students. An environment characterized by greater support and respect among all school agents is commonly associated with greater involvement on the part of students, which consequently translates into better academic and psychosocial outcomes. The importance of academic involvement applies to all students; however, it is especially relevant for students with CC [22]. 

A study by Lum et al. [29] showed that support at the school level is a factor associated with better school results and experiences for students with CC. A supportive behavior on the part of teachers is associated with better levels of school involvement. In turn, the adoption of school practices that promote student involvement can be a good strategy to support the more specific needs of students with CC. Regarding the study limitations, it is worth mentioning that the data were self-reported and had a higher number of responses from female participants, which may reflect some biases that must be considered. Furthermore, the study was carried out during the COVID-19 pandemic and in a fully online modality. However, it is a study that allows us to deepen the perspective of first-year university students with CC (representing a clinical population) and allows for comparisons with other studies carried out with the same population. Thus, this study contributes to the increase of knowledge about this specific population (i.e., first-year students with CC) and the type of barriers that these students feel in terms of their school participation in such an important and decisive period of their lives. In a future study, it could be interesting to compare the results of students with CC in the 1st and in the last year of the university in order to analyze the differences between the two moments.

## 5. Conclusions

Living with a CC tends to have a considerable impact on all aspects of an individual’s life [4]. The literature reports several pieces of evidence of the burden that CC can cause. This reinforces the importance of understanding how young people manage situations of stress and adversity, so that it is possible to move towards a clearer notion of the processes of coping and adaptation to the disease [58,59]. 

It is essential to consider that children and young people who show greater difficulty in accepting their health condition may show a less adjusted physical and psychological functioning [60], which reinforces their need for interventions in terms of mental health promotion and prevention. 

The literature suggests the existence of a relationship between chronic health conditions and worse academic and vocational outcomes [14,61,62]. The challenges associated with moving to higher education can be even greater when they are interconnected with the experience of a CC [11,15,18,20,21]. Therefore, it is important to explore the factors and barriers that can influence students with CC participation and academic success [22].

The literature also mentions the difficulties of young people with CC in the transition from pediatric to adult health care [63,64]. At the beginning of their academic career in higher education, such students are often on their own, outside their family home, managing their own health and well-being [17,19]. Therefore, it is a period that requires special health care and school monitoring. These results pinpoint powerful messages for health professionals, highlighting the gap and the vulnerability of health care when leaving adolescence (pediatric care) and entering adulthood (adult care). It is also valuable insight for policymakers in organizing care for university students with chronic health conditions.

## Figures and Tables

**Table 1:** Measures and variables under study.

Variables	Measures
Recent diagnosis (in the last two years)	Recoded variable: 0—No; 1—Yes (original variable: 1—at birth; 2—before entering school; 3—1st cycle; 4—2nd cycle; 5—3rd cycle; 6—high school; 7—recently (last two years)
Concerns	Recoded variable: 0—No; 1—Yes (original variable: 1—Several times a day; 2—Practically every day; 3—Several times a week; 4—Several times a month; 5—Rarely or never worried)
School physical environment as a barrier to participation and academic success	0—No; 1—Yes
People’s attitude towards CC as a barrier to participation and academic success	0—No; 1—Yes
Health condition as a barrier to participation and academic success	0—No; 1—Yes
Physical symptoms	Scale with five items (back pain, neck pain, headache, dizziness and stomach pain), on a five-point Likert scale (1—almost every day and 5—rarely or never). Minimum score of 5 and maximum of 25. The higher the value, the fewer physical symptoms. *α* = 0.76
Psychological symptoms	Scale with five items (nervousness, irritation or bad mood, sadness and fear), on a five-point Likert scale (1—almost every day and 5—rarely or never). Minimum score of 4 and maximum of 20). The higher the value, the fewer psychological symptoms. *α* = 0.86
Relationship with teachers	Scale adapted from Cantril [50], consisting of 11 steps, where the lowest step (0) corresponds to the worst quality of the relationship with teachers and the highest step (10) to the best quality of the relationship with teachers
Relationship with peers	Scale adapted from Cantril [50], consisting of 11 steps, where the lowest step (0) corresponds to the worst quality of the relationship with colleagues and the highest step (10) to the best quality of the relationship with peers
School participation affected by the CC	Scale with three items, which assess the impact that the chronic condition had on school participation (attendance, participation and academic success). *α* = 0.74 The variable was dichotomized with reference to affect or not to affect participation (No/Yes)

**Table 2:** Population characteristics and bivariate analysis of differences between students with and without recent diagnosis.

	*M* ± *SD* or % (*n*)			*p*
	Total (*n* = 207)	Recent Diagnosis No 71.3% (*n* = 127)	Recent Diagnosis Yes 28.7% (*n* = 50)	
Concerns ^2^				0.633
No	10.7 (22)	11.4 (14)	14.0 (7)	
Yes	89.3 (182)	88.6 (109)	86.0 (43)	
School physical environment as a barrier to participation and academic success ^2^				0.447
No	94.0 (172)	92.2 (106)	95.6 (43)	
Yes	6.0 (11)	7.8 (9)	4.4 (2)	
People’s attitude towards CC as a barrier to participation and academic success ^2^				0.961
No	86.9 (159)	87.0 (100)	86.7 (39)	
Yes	13.1 (24)	13.0 (15)	13.3 (6)	
Health condition as a barrier to participation and academic success ^2^				≤0.01
No	80.9 (148)	* 85.2 (98)	66.7 (30)	
Yes	19.1 (35)	14.8 (17)	* 33.3 (15)	
Physical symptoms ^1^	18.13 ± 4.66	17.98 ± 4.70	17.59 ± 4.67	0.934
Psychological symptoms ^1^	12.52 ± 4.46	12.59 ± 4.32	12.18 ± 4.53	0.634
Relationship with teachers ^1^	6.86 ± 1.91	6.99 ± 1.85	6.26 ± 2.00	0.592
Relationship with peers ^1^	7.38 ± 2.01	7.43 ± 1.89	6.98 ± 2.45	≤0.001
School participation affected by the CC ^1^	3.45 ± 0.90	3.23 ± 0.66	4.00 ± 1.21	≤0.001
No ^2^	76.3 (148)	* 87.0 (107)	52.0 (26)	
Yes ^2^	23.7 (46)	13.0 (16)	* 48.0 (24)	

^1^ Independent sample *t*-test; ^2^ chi-square. * Adjusted residuals > 1.96. Abbreviations: *M*, mean; *SD*, standard deviation.

**Table 3:** Bivariate analysis of differences between students with and without school participation affected by CC.

	*M* ± *SD* or % (*n*)		*p*
	School Participation Affected by the CC No 76.3% (*n* = 148)	School Participation Affected by the CC Yes 23.7% (*n* = 46)	
Recent diagnosis ^2^			≤0.001
No	* 80.5 (107)	40.0 (16)	
Yes	19.5 (26)	* 60.0 (24)	
Concerns ^2^			0.277
No	12.2 (18)	6.5 (3)	
Yes	87.8 (129)	93.5 (43)	
School physical environment as a barrier to participation and academic success ^2^			0.722
No	94.2 (130)	92.7 (38)	
Yes	5.8 (8)	7.3 (3)	
People’s attitude towards CC as a barrier to participation and academic success ^2^			≤0.001
No	* 94.2 (130)	61.0 (25)	
Yes	5.8 (8)	* 39.0 (16)	
Health condition as a barrier to participation and academic success ^2^			≤0.001
No	* 94.2 (130)	34.1 (14)	
Yes	5.8 (8)	* 65.9 (27)	
Physical symptoms ^1^	18.74 ± 4.33	16.00 ± 5.34	≤0.01
Psychological symptoms ^1^	13.14 ± 4.36	10.00 ± 4.25	0.941
Relationship with teachers ^1^	7.19 ± 1.75	5.87 ± 2.21	0.138
Relationship with peers ^1^	7.70 ± 1.82	6.35 ± 2.34	≤0.05

^1^ Independent sample *t*-test; ^2^ chi-square. * Adjusted residuals > 1.96. Abbreviations: *M*, mean; *SD*, standard deviation.

**Table 4:** Linear regression model of variables for the study of school participation affected by the CC. Predictors: Time of diagnosis, barriers to school participation (health condition, people’s attitude towards CC and school physical environment), physical and mental health (symptoms and concerns), and school-related variables (relationship with teachers and peers).

	Non-Standardized Coefficient		Standardized Coefficient	*t*
	B	Standard Error	*β*	
Recent diagnosis	0.39	0.12	0.19 **	3.21
Physical symptoms	0.01	0.01	0.07	1.10
Psychological symptoms	−0.03	0.01	−0.15 *	−2.15
Concerns	−0.28	0.16	−0.11	−1.73
School physical environment as a barrier to participation and academic success	−0.19	0.20	−0.06	−0.96
People’s attitude towards the chronic condition as a barrier to participation and academic success	0.55	0.16	0.21 ***	3.44
Health condition as a barrier to participation and academic success	1.05	0.14	0.45 ***	7.34
Relationship with teachers	−0.08	0.03	−0.16 **	−2.55
Relationship with peers	−0.06	0.03	−0.14 *	−2.20

The results were adjusted to age and gender. The variables were entered using the “enter” mode. * *p* ≤ 0.05; ** *p* ≤ 0.01; *** *p* ≤ 0.001 *R*^2^ = 0.55.

## Data Availability

The data that support the findings of this study are available from the corresponding author upon reasonable request.
